# Supplementary material for: Association of the awareness of the epidemic, mental health status with mobile phone screen use time in Chinese college students during COVID-19 isolation and control
Source: PLOS Glob Public Health. 2023 Feb 8;3(2):e0001259. doi: 10.1371/journal.pgph.0001259 (PMC10021596; doi:10.1371/journal.pgph.0001259)
Supplement: Attachment — Submitted filename: PLOS ONE-Response to Reviewers.docx [file pgph.0001259.s002.docx]

Dear Editors (Prof.Muhammad Fawad Rasool) and reviewers:

Thank you for your precious comments and advice. Those comments are all valuable and very helpful for revising and improving our paper, as well as the important guiding significance to our researches. We have studied comments carefully and have made correction which we hope meet with approval. Revised portion are marked in red in the paper. The main corrections in the paper and the responds to the reviewer’s comments are as flowing:

Response to the reviewer's comments:

Reviewer #1:

Thank you for your summary. We really appreciate your efforts in reviewing our manuscript. We have revised the manuscript accordingly. Our point-by-point responses are detailed below.

1. Manuscript has a date modified printed on it April 2021 while date of survey claimed in the script is January 9-12 2022 (need to checked)
   **Response:** Thank you for underlining this deficiency. This section was revised and modified according to the information showed in the work suggested by the reviewer .
2. The questions regarding transmission route and protective measures supposed to be answered with multiple options, but authors reported them as independent options with no overlapping response. This need clarity, the method through which the questions were put in front of respondents.

**Response:** We are very grateful for your comments. Questions about the transmission route of the epidemic and protective measures should be designed as overlapping options, which can better reflect college students' knowledge and understanding of the epidemic.we will pay special attention to this aspect in future research work.The questionnaires in this study were designed through the Questionnaire Star platform, and a QR code was generated, which was forwarded through the WeChat group of each class. Students can fill in after scanning the code. The main information of the questionnaire includes general information, knowledge of the epidemic situation and mental health related issues.

3. Percentages reported in 2.3 are presented with mixed decimal places. Few (prevalence of stress 21.79%, mild (21.9%)). The percentages must be presented uniformly, I believe single decimal place is enough.
   **Response:** Thank you for the suggestion. We have revised the information required as explained above and has been marked in red (Lines 112-118, page 4；Lines 143, page 5).
4. The section 2.4, Table 1 expresses the scores for stress, anxiety and depression by using mean±sd. I wonder, whether the normality of data was tested?, as very high standard deviations suggest that data was deviating from normality. If so, in this case the data is better to be presented by median and interquartile range i.e. median (Q1 – Q3). The test for comparison will also change (Kruskal Wallis test is suggested) also need to have a post hoc analysis (Mann Whitney U if Kruskal Wallis and Tukey’s or Tamhane’s in case normality is in the data and ANOVA is applicable)
   **Response:**We are grateful for the suggestion. As suggested by the reviewer, we have revised more details of the section 2.4, Table 1 expresses the scores for stress, anxiety and depression.We tested the data for normality and found it to be skewed.Therefore,the data is be presented by median and interquartile range i.e. median (Q1 – Q3).The test for comparison using Kruskal Wallis test.
5. The scatter diagram (section 2.5, Figure 3) is presenting relation between time on mobile per day and DASS 21 score (sub items). It is interesting to observe that many scores go beyond 21(the maximum possible score through 7 items, scored between 0-3). Also the time spent per day exceeds 20 hours (beyond reality but still possible). The author need to recheck their score calculated for stress, anxiety and depression as none can exceed 21.
   **Response:** We are extremely grateful to you for pointing out this problem.Now we revise the following according to your comments :In this study, the DASS-21 scale was used to evaluate the psychological state of college students in the first week of the survey. Each item in the scale is scored between 0 and 3 points, with "0" for "never", "1" for "sometimes", "2" for "often", "3" for "always", the total scores of each subscale multiplied by 2 is the final score. According to the grading standard of each subscale of DASS-21, it can be divided into normal, mild, moderate, severe and extreme, and the higher the level, the more serious the negative emotion.

Tab.1 Standard of grades of each DASS-21 subscale

| Grading | depression | anxiety | stress |
| --- | --- | --- | --- |
| normal | 0～9 | 0～7 | 0～14 |
| mild | 10～13 | 8～9 | 15～18 |
| moderate | 14～20 | 10～14 | 19～25 |
| severe | 21～27 | 15～19 | 26～33 |
| extreme | 28+ | 20+ | 34+ |

Reviewer #2:

Thank you for your careful review. We are very sorry for the mistakes in this manuscript and inconvenience they caused in your reading. The manuscript has been thoroughly revised and rewritten by a native English speaker , so we hope it can meet the journal’s standard.

2. 1.Abstract need be revised again and should be simple and have clear meanings.Graphs are not very clear, I think it should be improved more.

**Response:** Thank you for the suggestion. We have revised the abstract section and some graphs of the article and used red markers.

3. There seems to a confusion regarding the results as the authors says that long mobile screen users know more about the transmission and prevention of SARS virus but at the same time data indicates more anxiety and depression.

**Response:** We further looked to see if knowledge of the spread and prevention of the outbreak had an effect in the fact that prolonged screen time led to more psychological problems, so we conducted a mediation effect analysis.The mediation effect analysis has not found that it has played an important role for the time being, so there is no obvious evidence to prove that the knowledge of epidemic spread and prevention has an impact on the long-term mobile phone screen use time leading to more psychological problems;We think there may be two reasons: 1. A larger sample size is needed; 2. The knowledge of epidemic spread and prevention has little influence on the relationship between mobile phone screen time and mental health, and is only a part of its influencing factors.
